# Poly[diimidazole-μ_4_-oxalato-μ_2_-oxalato-dicopper(II)]

**DOI:** 10.1107/S1600536811015777

**Published:** 2011-05-07

**Authors:** Zhu-Nian Jin, Hong Lin

**Affiliations:** a Jinhua Professional Technical College, No. 1188 Wuzhou Street, Jinhua, Zhejiang 321007, People’s Republic of China

## Abstract

The title compound, [Cu_2_(C_2_O_4_)_2_(C_3_H_4_N_2_)_2_]_*n*_, was obtained as an unexpected product under hydro­thermal conditions. The Cu^II^ atom is in a Jahn–Teller-distorted octa­hedral environment formed by one imidazole N atom and five O atoms from three oxalate anions. The two independent oxalate anions are situated on centres of inversion and coordinate to the Cu^II^ atom in two different modes, *viz*. bidentate and monodentate. The bidentate anions bridge two Cu^II^ atoms, whereas the monodentate anions bridge four Cu^II^ atoms, leading to a layered arrangement parallel to (100). These layers are further linked into a final three-dimensional network structure *via* inter­molecular N—H⋯O hydrogen bonds. The title compound is isotypic with the Zn analogue.

## Related literature

For background to oxalates, see: Ghosh *et al.* (2004 ▶); Ye & Lin (2010 ▶). For the isotypic Zn analogue, see: Lu *et al.* (2005 ▶).
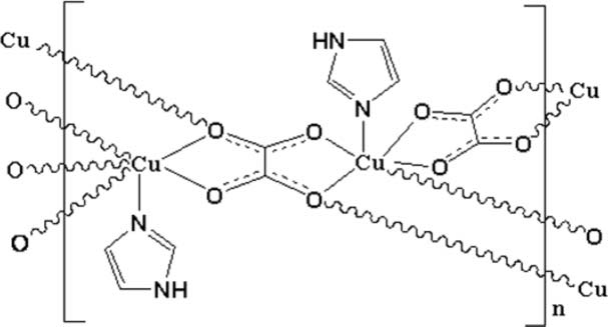


## Experimental

### 

#### Crystal data

[Cu_2_(C_2_O_4_)_2_(C_3_H_4_N_2_)_2_]
*M* *_r_* = 439.28
Monoclinic, 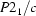

*a* = 8.3367 (4) Å
*b* = 9.3131 (5) Å
*c* = 8.4838 (5) Å
β = 92.352 (3)°
*V* = 658.13 (6) Å^3^
*Z* = 2
Mo *K*α radiation
μ = 3.29 mm^−1^
*T* = 296 K
0.28 × 0.18 × 0.06 mm

#### Data collection

Bruker APEXII CCD diffractometer
Absorption correction: multi-scan (*SADABS*; Sheldrick, 1996 ▶) *T*_min_ = 0.497, *T*_max_ = 0.821
10313 measured reflections
1511 independent reflections
1362 reflections with *I* > 2σ(*I*)
*R*_int_ = 0.028

#### Refinement

*R*[*F* ^2^ > 2σ(*F* ^2^)] = 0.022
*wR*(*F* ^2^) = 0.062
*S* = 1.09
1511 reflections
109 parameters
H-atom parameters constrained
Δρ_max_ = 0.49 e Å^−3^
Δρ_min_ = −0.42 e Å^−3^

### 

Data collection: *APEX2* (Bruker, 2006 ▶); cell refinement: *SAINT* (Bruker, 2006 ▶); data reduction: *SAINT*; program(s) used to solve structure: *SHELXS97* (Sheldrick, 2008 ▶); program(s) used to refine structure: *SHELXL97* (Sheldrick, 2008 ▶); molecular graphics: *DIAMOND* (Brandenburg, 2006 ▶); software used to prepare material for publication: *SHELXTL* (Sheldrick, 2008 ▶).

## Supplementary Material

Crystal structure: contains datablocks I, global. DOI: 10.1107/S1600536811015777/wm2473sup1.cif

Structure factors: contains datablocks I. DOI: 10.1107/S1600536811015777/wm2473Isup2.hkl

Additional supplementary materials:  crystallographic information; 3D view; checkCIF report

## Figures and Tables

**Table 1:** Selected bond lengths (Å)

Cu1—N1	1.9624 (18)
Cu1—O3	1.9713 (13)
Cu1—O2^i^	1.9960 (14)
Cu1—O1	2.0016 (13)
Cu1—O4	2.3536 (14)
Cu1—O4^ii^	2.512 (1)

Symmetry codes: (i) 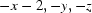; (ii) 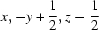.

**Table 2:** Hydrogen-bond geometry (Å, °)

*D*—H⋯*A*	*D*—H	H⋯*A*	*D*⋯*A*	*D*—H⋯*A*
N2—H2*A*⋯O2^iii^	0.86	2.00	2.841 (2)	167

Symmetry code: (iii) 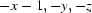.

**Fig. 1.**
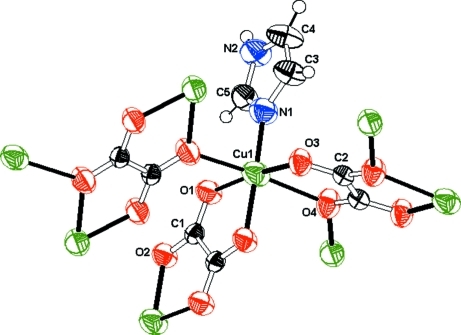
Part of the structure of Cu2(C2O4)2(C3N2H4)2. Displacement ellipsoids are drawn at the 30% probability level.

**Fig. 2.**
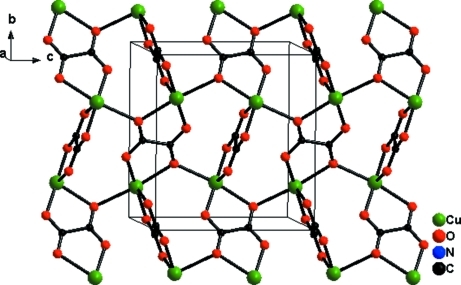
The two-dimensional layer constructed by bridging oxalate anions. Imidazole ligands are omitted for clarity.

**Fig. 3.**
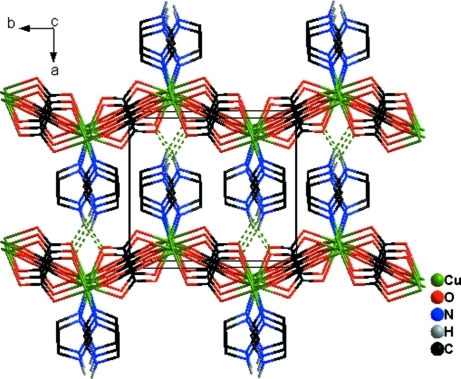
The three-dimensional network in Cu2(C2O4)2(C3N2H4)2. The N—H···O hydrogen bond interactions are depicted by dashed lines.

## supplementary crystallographic information

### Comment

Oxalates can represent one of the end-products of the degradation of some organic ligands, under both oxidative and non-oxidative conditions (Ghosh *et al.*, 2004). For example, we reported an oxalate compound with a three-dimensional structure, which was constructed by decomposition of 2-carboxymethylsulfanyl nicotinic acid (Ye *et al.*, 2010). Herein, we report a new polymeric oxalate compound, Cu_2_(C_2_O_4_)_2_(C_3_N_2_H_4_)_2_, (I), which is isotypic with Zn_2_(C_2_O_4_)_2_(C_3_N_2_H_4_)_2_ (Lu *et al.*, 2005).

A view on the molecular structure of compound (I) is given in Fig. 1. The Cu^II^ atoms are each in a Jahn-Teller distorted coordination by one nitrogen atom from imidazole and five oxygen atoms from three oxalate groups. The oxalate anions adopt two different coordination modes: one adopts a chelate bis-bidentate linkage, the other adopts a chelate and bridging bis-bidentate linkage (Fig. 1). As shown in Fig. 2, the oxalate anions connect the Cu^II^ atoms into a two dimensional layer along the *bc* plane, and are further linked into a three-dimensional network structure by N—H···O hydrogen bonds (Fig. 3).

### Experimental

A mixture of 2-carboxymethylsulfanyl nicotinic acid (0.086 g, 0.40 mmol), CuCl_2_^.^2H_2_O (0.068 g, 0.40 mmol), and imidazole (0.054 g, 0.80 mmol) in CH_3_CH_2_OH (2 ml)/H_2_O (16 ml) was placed in a 25 ml Teflon-lined stainless steel reactor and heated at 383 K for 24 h, and then cooled to room temperature over a period of 24 h. Green crystals suitable for X-ray analysis were obtained.

### Refinement

The H-atoms were positioned geometrically and included in the refinement using a riding model [C—H 0.93Å and *U*_iso_(H) = 1.2*U*_eq_(C); N—H 0.86 Å; and *U*_iso_(H) = 1.2*U*_eq_(N)].

### Crystal data

**Table:**

[Cu_2_(C_2_O_4_)_2_(C_3_H_4_N_2_)_2_]	*F*(000) = 436
*M**_r_* = 439.28	*D*_x_ = 2.217 Mg m^−^^3^
Monoclinic, *P*2_1_/*c*	Mo *K*α radiation, λ = 0.71073 Å
Hall symbol: -P 2ybc	Cell parameters from 4365 reflections
*a* = 8.3367 (4) Å	θ = 2.5–27.6°
*b* = 9.3131 (5) Å	µ = 3.29 mm^−^^1^
*c* = 8.4838 (5) Å	*T* = 296 K
β = 92.352 (3)°	Block, green
*V* = 658.13 (6) Å^3^	0.28 × 0.18 × 0.06 mm
*Z* = 2	

### Data collection

**Table:**

Bruker APEXII CCD diffractometer	1511 independent reflections
Radiation source: fine-focus sealed tube	1362 reflections with *I* > 2σ(*I*)
graphite	*R*_int_ = 0.028
ω scans	θ_max_ = 27.6°, θ_min_ = 2.5°
Absorption correction: multi-scan (*SADABS*; Sheldrick, 1996)	*h* = −10→9
*T*_min_ = 0.497, *T*_max_ = 0.821	*k* = −12→12
10313 measured reflections	*l* = −11→10

### Refinement

**Table:**

Refinement on *F*^2^	Primary atom site location: structure-invariant direct methods
Least-squares matrix: full	Secondary atom site location: difference Fourier map
*R*[*F*^2^ > 2σ(*F*^2^)] = 0.022	Hydrogen site location: inferred from neighbouring sites
*wR*(*F*^2^) = 0.062	H-atom parameters constrained
*S* = 1.09	*w* = 1/[σ^2^(*F*_o_^2^) + (0.0335*P*)^2^ + 0.3353*P*] where *P* = (*F*_o_^2^ + 2*F*_c_^2^)/3
1511 reflections	(Δ/σ)_max_ = 0.001
109 parameters	Δρ_max_ = 0.49 e Å^−^^3^
0 restraints	Δρ_min_ = −0.42 e Å^−^^3^

### Special details

**Table:**

Geometry. All e.s.d.'s (except the e.s.d. in the dihedral angle between two l.s. planes) are estimated using the full covariance matrix. The cell e.s.d.'s are taken into account individually in the estimation of e.s.d.'s in distances, angles and torsion angles; correlations between e.s.d.'s in cell parameters are only used when they are defined by crystal symmetry. An approximate (isotropic) treatment of cell e.s.d.'s is used for estimating e.s.d.'s involving l.s. planes.
Refinement. Refinement of *F*^2^ against ALL reflections. The weighted *R*-factor *wR* and goodness of fit *S* are based on *F*^2^, conventional *R*-factors *R* are based on *F*, with *F* set to zero for negative *F*^2^. The threshold expression of *F*^2^ > σ(*F*^2^) is used only for calculating *R*-factors(gt) *etc*. and is not relevant to the choice of reflections for refinement. *R*-factors based on *F*^2^ are statistically about twice as large as those based on *F*, and *R*- factors based on ALL data will be even larger.

### Fractional atomic coordinates and isotropic or equivalent isotropic displacement parameters (Å^2^)

**Table:**

	*x*	*y*	*z*	*U*_iso_*/*U*_eq_	
Cu1	−0.87721 (3)	0.23821 (2)	−0.10302 (3)	0.02435 (10)	
O1	−0.80090 (16)	0.04677 (14)	−0.02324 (17)	0.0263 (3)	
O2	−0.90639 (16)	−0.15701 (14)	0.06412 (17)	0.0265 (3)	
O3	−0.97708 (18)	0.41685 (14)	−0.18398 (16)	0.0277 (3)	
O4	−0.91189 (18)	0.37282 (15)	0.12789 (17)	0.0296 (3)	
N1	−0.6552 (2)	0.30160 (19)	−0.1281 (2)	0.0294 (4)	
N2	−0.3925 (2)	0.2996 (3)	−0.1154 (3)	0.0450 (5)	
H2A	−0.2956	0.2704	−0.0975	0.054*	
C1	−0.9154 (2)	−0.03131 (19)	0.0114 (2)	0.0214 (4)	
C2	−1.0193 (2)	0.51365 (19)	−0.0895 (2)	0.0225 (4)	
C3	−0.5993 (3)	0.4276 (3)	−0.1902 (3)	0.0411 (5)	
H3A	−0.6631	0.5019	−0.2305	0.049*	
C4	−0.4374 (3)	0.4264 (3)	−0.1836 (3)	0.0483 (6)	
H4A	−0.3699	0.4981	−0.2186	0.058*	
C5	−0.5252 (3)	0.2292 (2)	−0.0814 (3)	0.0400 (6)	
H5A	−0.5266	0.1404	−0.0313	0.048*	

### Atomic displacement parameters (Å^2^)

**Table:**

	*U*^11^	*U*^22^	*U*^33^	*U*^12^	*U*^13^	*U*^23^
Cu1	0.02007 (15)	0.01856 (14)	0.03460 (17)	0.00096 (8)	0.00343 (10)	0.00411 (9)
O1	0.0212 (7)	0.0228 (6)	0.0351 (8)	−0.0002 (5)	0.0017 (6)	0.0042 (6)
O2	0.0215 (7)	0.0203 (6)	0.0377 (8)	0.0020 (5)	0.0023 (6)	0.0040 (5)
O3	0.0356 (8)	0.0206 (6)	0.0269 (7)	0.0042 (5)	0.0015 (6)	0.0004 (5)
O4	0.0369 (8)	0.0235 (7)	0.0284 (7)	0.0072 (6)	0.0001 (6)	0.0016 (5)
N1	0.0244 (8)	0.0261 (8)	0.0379 (10)	−0.0009 (7)	0.0034 (7)	0.0014 (7)
N2	0.0216 (10)	0.0462 (12)	0.0670 (15)	0.0008 (8)	0.0001 (9)	0.0017 (10)
C1	0.0204 (9)	0.0217 (8)	0.0222 (9)	0.0022 (7)	0.0013 (7)	−0.0013 (7)
C2	0.0224 (9)	0.0214 (8)	0.0238 (10)	−0.0019 (7)	0.0008 (7)	0.0019 (7)
C3	0.0339 (12)	0.0401 (12)	0.0490 (14)	−0.0052 (10)	−0.0025 (10)	0.0143 (10)
C4	0.0334 (12)	0.0551 (15)	0.0561 (16)	−0.0138 (11)	−0.0001 (11)	0.0164 (13)
C5	0.0272 (12)	0.0303 (11)	0.0624 (16)	0.0021 (8)	0.0015 (11)	0.0043 (10)

### Geometric parameters (Å, °)

**Table:**

Cu1—N1	1.9624 (18)	N1—C3	1.375 (3)
Cu1—O3	1.9713 (13)	N2—C5	1.328 (3)
Cu1—O2^i^	1.9960 (14)	N2—C4	1.361 (3)
Cu1—O1	2.0016 (13)	N2—H2A	0.8600
Cu1—O4	2.3536 (14)	C1—C1^i^	1.532 (4)
Cu1—O4^ii^	2.512 (1)	C2—O4^iii^	1.240 (2)
O1—C1	1.245 (2)	C2—C2^iii^	1.560 (4)
O2—C1	1.254 (2)	C3—C4	1.348 (3)
O2—Cu1^i^	1.9960 (14)	C3—H3A	0.9300
O3—C2	1.266 (2)	C4—H4A	0.9300
O4—C2^iii^	1.240 (2)	C5—H5A	0.9300
N1—C5	1.323 (3)		
			
N1—Cu1—O3	95.45 (7)	C3—N1—Cu1	129.37 (16)
N1—Cu1—O2^i^	174.07 (6)	C5—N2—C4	107.7 (2)
O3—Cu1—O2^i^	90.33 (6)	C5—N2—H2A	126.2
N1—Cu1—O1	90.95 (6)	C4—N2—H2A	126.2
O3—Cu1—O1	173.43 (6)	O1—C1—O2	126.46 (17)
O2^i^—Cu1—O1	83.31 (5)	O1—C1—C1^i^	117.3 (2)
N1—Cu1—O4	94.51 (7)	O2—C1—C1^i^	116.2 (2)
O3—Cu1—O4	77.05 (5)	O4^iii^—C2—O3	125.35 (17)
O2^i^—Cu1—O4	85.50 (6)	O4^iii^—C2—C2^iii^	118.0 (2)
O1—Cu1—O4	103.96 (5)	O3—C2—C2^iii^	116.7 (2)
O1—Cu1—O4^ii^	87.92 (5)	C4—C3—N1	109.4 (2)
O4—Cu1—O4^ii^	164.20 (6)	C4—C3—H3A	125.3
O3—Cu1—O4^ii^	89.98 (5)	N1—C3—H3A	125.3
N1—Cu1—O4^ii^	95.69 (6)	C3—C4—N2	106.4 (2)
C1—O1—Cu1	111.35 (12)	C3—C4—H4A	126.8
C1—O2—Cu1^i^	111.80 (12)	N2—C4—H4A	126.8
C2—O3—Cu1	120.32 (12)	N1—C5—N2	111.3 (2)
C2^iii^—O4—Cu1	107.87 (12)	N1—C5—H5A	124.4
C5—N1—C3	105.23 (19)	N2—C5—H5A	124.4
C5—N1—Cu1	125.38 (16)		
			
N1—Cu1—O1—C1	178.87 (13)	O2^i^—Cu1—N1—C3	−170.1 (6)
O3—Cu1—O1—C1	−14.0 (6)	O1—Cu1—N1—C3	175.6 (2)
O2^i^—Cu1—O1—C1	0.34 (13)	O4—Cu1—N1—C3	−80.3 (2)
O4—Cu1—O1—C1	83.99 (13)	Cu1—O1—C1—O2	179.88 (16)
N1—Cu1—O3—C2	−96.29 (15)	Cu1—O1—C1—C1^i^	−0.4 (3)
O2^i^—Cu1—O3—C2	82.40 (15)	Cu1^i^—O2—C1—O1	179.67 (16)
O1—Cu1—O3—C2	96.7 (5)	Cu1^i^—O2—C1—C1^i^	0.0 (2)
O4—Cu1—O3—C2	−2.90 (14)	Cu1—O3—C2—O4^iii^	−177.21 (15)
N1—Cu1—O4—C2^iii^	97.23 (13)	Cu1—O3—C2—C2^iii^	2.8 (3)
O3—Cu1—O4—C2^iii^	2.66 (13)	C5—N1—C3—C4	1.7 (3)
O2^i^—Cu1—O4—C2^iii^	−88.72 (13)	Cu1—N1—C3—C4	179.68 (18)
O1—Cu1—O4—C2^iii^	−170.67 (12)	N1—C3—C4—N2	−0.6 (3)
O3—Cu1—N1—C5	174.7 (2)	C5—N2—C4—C3	−0.7 (3)
O2^i^—Cu1—N1—C5	7.6 (8)	C3—N1—C5—N2	−2.1 (3)
O1—Cu1—N1—C5	−6.7 (2)	Cu1—N1—C5—N2	179.75 (17)
O4—Cu1—N1—C5	97.3 (2)	C4—N2—C5—N1	1.8 (3)
O3—Cu1—N1—C3	−2.9 (2)		

Symmetry codes: (i) −*x*−2, −*y*, −*z*; (ii) *x*, −*y*+1/2, *z*−1/2; (iii) −*x*−2, −*y*+1, −*z*.

### Hydrogen-bond geometry (Å, °)

**Table:**

*D*—H···*A*	*D*—H	H···*A*	*D*···*A*	*D*—H···*A*
N2—H2A···O2^iv^	0.86	2.00	2.841 (2)	167

Symmetry codes: (iv) −*x*−1, −*y*, −*z*.
